# Predicting gene function using similarity learning

**DOI:** 10.1186/1471-2164-14-S4-S4

**Published:** 2013-10-01

**Authors:** Tu Minh Phuong, Ngo Phuong Nhung

**Affiliations:** 1 Department of Computer Science, Posts & Telecommunications Institute of Technology, Hanoi, Viet Nam; 2 KRDB Research Center, Free University of Bolzano, Bolzano, Italy

## Abstract

**Background:**

Computational methods that make use of heterogeneous biological datasets to predict gene function provide a cost-effective and rapid way for annotating genomes. A common framework shared by many such methods is to construct a combined functional association network from multiple networks representing different sources of data, and use this combined network as input to network-based or kernel-based learning algorithms. In these methods, a key factor contributing to the prediction accuracy is the network quality, which is the ability of the network to reflect the functional relatedness of gene pairs. To improve the network quality, a large effort has been spent on developing methods for network integration. These methods, however, produce networks, which then remain unchanged, and nearly no effort has been made to optimize the networks after their construction.

**Results:**

Here, we propose an alternative method to improve the network quality. The proposed method takes as input a combined network produced by an existing network integration algorithm, and reconstructs this network to better represent the co-functionality relationships between gene pairs. At the core of the method is a learning algorithm that can learn a measure of functional similarity between genes, which we then use to reconstruct the input network. In experiments with yeast and human, the proposed method produced improved networks and achieved more accurate results than two other leading gene function prediction approaches.

**Conclusions:**

The results show that it is possible to improve the accuracy of network-based gene function prediction methods by optimizing combined networks with appropriate similarity measures learned from data. The proposed learning procedure can handle noisy training data and scales well to large genomes.

## Background

The increasing number of sequenced genomes makes it important to develop methods that can assign functions to newly discovered genes in a timely and cost-effective manner. Traditional laboratory methods, while accurate and reliable, would require enormous effort and time to identify functions for every gene. Computational approaches that utilize diverse biological datasets to generate automated predictions are useful in this situation as they can guide laboratory experiments and facilitate more rapid annotation of genomes.

Existing computational approaches to gene function prediction have relied on a variety of genomic and proteomic data. Exploiting the similarities between DNA or protein sequences to infer gene function was the first approach tested and has been the most widely used approach to date. Later, the usefulness of other types of genomic and proteomic data in this problem is also proved. Researchers have used microarray expression data [1], protein 3D structures [2], protein domain configuration [3], protein-protein interaction networks [4], and phylogenetic profiles [5] to predict functions of genes. Recently, inferring gene function simultaneously from different types of biological data has been shown to deliver more accurate predictions and has attracted considerable research interests [6-16].

Many methods for inferring functions of genes from heterogeneous datasets share a common framework in which a functional association between genes is first constructed and then used as input for learning algorithms. A functional association can be represented as a network with nodes corresponding to genes and edges representing the co-functionalities of gene pairs. In such a network, each edge is usually assigned a weight representing the strength of the co-functionality relationship between the gene pair. A network of this kind is typical constructed in two steps. First, each dataset is used to create an individual network that captures the co-functionality of gene pairs, as implied by this dataset. For vectorial data, one can calculate edge weights as the similarity scores between genes using appropriate similarity metrics, for example the Pearson correlation coefficient, and then form the networks by means of neighboring node connections. Data already given in forms of networks, for example protein-protein interactions, are used directly. The second step constructs a single combined association network by integrating the individual ones. A strategy commonly used in this step is to form the combined network as a weighted sum of individual ones. Here, each network is weighted according to its usefulness in predicting annotations for a group of genes that share a known specific function. Previous studies have used various regression or other learning based algorithms to estimate network weights.

Given a functional association network, the next step is to use this network to propagate functional labels from a group of annotated genes to other genes. There are two main types of approaches for this step. Approaches of the first type create a kernel function from the co-functionality relationships encoded in the network and use this kernel with kernel-based classification algorithms [8,9,17]. In such approaches, genes with known annotations serve as labeled examples for training. Approaches of the second type use graph-based algorithms, which propagate labels from annotated genes to other genes based on graph proximity. Methods in this group range from simple nearest neighbor counting algorithms [16], to more sophisticated statistical methods such as graph-based semi-supervised learning algorithms [9], and Markov random fields [18] (see [19] for a more complete list of methods). On a number of benchmark datasets, graph-based and kernel-based approaches have shown comparable prediction accuracy, but graph-based approaches are generally faster [11,20].

The prediction accuracy of both graph-based and kernel-based approaches largely depends on the ability of the network to capture the functional associations between genes. To improve the network quality, previous studies have focused on improving the integration step, or more precisely, on learning optimized weights for individual networks, and little effort has been applied toward improving the combined networks after they are constructed.

In this study, which is an extension of our previous work [22], we assume that the network integration step is already done and focus on optimizing the produced network. Given a combined network and a set of annotated genes that serve as training examples, we present a method for learning networks of improved quality. This is done in two steps: in the first step, the method learns a measure of similarity between pairs of genes; in the second step, the method reweights the network's edges using the similarity measure it just learned. Here, we are inspired by previous work in ranking and multimedia retrieval domains which improves search results by learning a measure of semantic similarity from online datasets and using it to rank multimedia objects [23,24]. In learning, the algorithm iteratively updates a similarity function so that it gives higher scores to pairs of similar objects and lower scores to dissimilar or randomized pairs. When learning ends, semantically related objects are more likely to get higher similarity scores. Once the similarity scores are learned, we use them to re-weight the edges of the input network.

In predicting gene function, discriminative learning algorithms are challenged by the small number of positive genes (genes annotated to a given category) for many categories, which is known as the problem of learning with unbalanced data. This problem is less critical for similarity learning methods because they tend to assume a weaker form of supervision than in classification, in which no labels are provided. Moreover, whenever genes annotated to functional categories are available, the category labels induce a notion of similarity across pairs, and this similarity can easily be incorporated into the learning process. Thus, similarity learning offers a more flexible framework than classification algorithms and can handle problems associated with unbalanced data in a natural way. Another challenge for gene function prediction algorithms is speed, especially when assigning functions in large genomes comprising tens of thousands of genes. Here, we use a learning algorithm that scales to the large genome size. The algorithm achieves computational efficiency due to several factors. It exploits sparse representations of genes when computing similarity, it does not require a similarity function to be symmetric or positive, and it is based on an online passive-aggressive algorithm that is known to converge quickly after being presented with only a handful of training examples.

We evaluated the effectiveness of the method (which we call *Similarity Learning of Association Networks *[SLAN]) in predicting Gene Ontology (GO) functional categories of genes in yeast and human using several datasets. As shown by the results, SLAN was able to learn networks that yielded more accurate predictions, as compared to predictions produced by *fixed *networks. In a comparison with two state-of-the-art gene function prediction methods, SLAN achieved higher prediction accuracy even when given as input a combined network produced by a simple integration method. The results also show that the method scales well with the number of genes.

## Methods

The proposed method predicts gene function using the following steps: (i) learning a measure of functional similarity between gene pairs; (ii) using this similarity measure to form new association networks; and (iii) inferring functions of genes from the new networks. In the following sections, we first describe the algorithm that learns similarity functions from data, and the method for selecting training samples. Next, we describe how new networks are constructed using the learned functions. Then, we give a brief review of the algorithm that predicts gene function from reconstructed networks. Finally, we describe the datasets and input networks used in our experiments.

### The similarity learning algorithm

Assume there are *n *genes *g*_1_, ..., *g_n_*, the first *d *genes of which have annotations in forms of GO terms, where each GO term corresponds to a category of gene function, and the remaining genes are new, the annotations of which are unknown and to be predicted. We also assume we are provided as input a functional association network with *n *nodes; each node corresponds to a gene, and each (weighted) edge represents the evidence of a functional association between the gene pair. Each edge connecting gene *g_i _*and gene *g_j _*is assigned a positive weight *a_ij _*, which shows the strength of this association. Such a network can be constructed from heterogeneous datasets using network integration methods like the one presented in [9,10]. Using the set of *d *genes with known annotations as training data, our method estimates a measure of semantic similarity that reflects the functional relatedness between gene pairs. With the learned similarity measure, we reconstruct the input network and use the new network to predict function for new genes. Note that, in the learning phase, the method has access only to a fragment of network comprising *d *annotated genes, while in prediction it uses the full network of *n *genes.

The similarity learning algorithm we use in this study requires input objects be represented as vectors of real-valued features. To transform genes into vectors of real numbers, we apply a feature map $\Phi \left(.\right)\in {ℜ}^{d}$, which represents gene *g_l _*as the following column vector

(1) $$\Phi \left(g_{l}\right)={\left\{a_{l1},\ldots ,a_{ld}\right\}}^{T},$$

where *a_li _*for *i *= 1,..,*d *are edge weights taken from the input network. Intuitively, each gene is represented by its similarities to the *d *annotated genes, according to the given network.

Now, let training signals be given in the form of a set *P *of gene triplets (*g*, *g*^+^, *g*^-^), where genes *g *and *g*^+ ^are in a stronger functional association than genes *g *and *g*^-^. The goal is to learn a similarity function *S*(.,.) that assigns higher similarity scores for pairs of more functionally relevant genes, that is *S*(*g*, *g*^+^) >*S*(*g*, *g*^-^), ∀(*g*, *g *^+^, *g*^-^).

Here, we adopt the learning algorithm originally proposed for image search applications [23]. The algorithm learns a similarity function that has the bilinear form:

(2) $$S_{W}\left(g_{i},g_{j}\right)=\Phi^{T}\left(g_{i}\right)W\Phi \left(g_{j}\right),$$

where $W\in {ℜ}^{dxd}$ is a parameter matrix. It is important to note that, in practice, a widely used preprocessing step is to sparsify the association network by keeping only *k *strongest connections for each gene (*k *<<*d*). In such a sparse representation, only *k *elements of feature vector Φ(*g_i_*) are non-zero. Therefore, the computation of function *S_W _*has complexity O(*k*^2^) regardless of *d*. This property makes the computation of the similarity function efficient when *d *is large.

In the learning phase, the algorithm estimates a parameter matrix *W *such that gene pairs in stronger functional associations are assigned higher scores. Specifically, for all triplets (*g*, *g *^+^, *g*^-^) ∈*P *, the algorithm seeks to find a matrix *W *such that *S*(*g*, *g*^+^) is larger than *S*(*g*, *g*^-^) with a safety margin of 1:

(3) $$S_{W}\left(g,g^{+}\right)-S_{W}\left(g,g^{-}\right)\geq 1,$$

For triplet (*g*, *g*^+^, *g*^-^) the algorithm computes the following hinge loss function:

(4) $$l_{W}\left(g,\;g^{+},g^{-}\right)=\text{max}\left(1-S_{W}\left(g,\;g^{+}\right)+S_{W}\left(g,\;g^{-}\right),0\right),$$

When the safety margin (3) is violated, this loss function is positive, making a penalty to the objective function. The algorithm then tries to minimize a training objective function that accumulates losses over all training data:

(5) $$L_{W}=\underset{\left(g,g^{+},g^{-}\right)\in P}{\sum }l_{W}\left(g,g^{+},g^{-}\right),$$

This objective function is minimized by applying the Passive-Aggressive algorithm [24] iteratively over training triplets. First, the algorithm initializes *W *to some matrix *W*^0 ^(in our experiments, *W*^0 ^was initialized to an identity matrix). Then, in each iteration, the algorithm selects at random a triplet (*g*, *g*^+^, *g*^-^) ∈ *P *and computes the hinge loss according to (4). If *l_W _*(*g*, *g*^+^, *g*^-^) = 0, or, equivalently, $S_{W}\left(g,g^{+}\right)-S_{W}\left(g,g^{-}\right)\geq 1$, no update is made. Otherwise, it solves the following convex problem with a soft margin:

(6) $$W^{i}=\underset{w}{\text{arg}\;\text{min}}\;∥\;W-W^{i-1}{{∥}^{2}}_{Frob}+\alpha \xi ,$$

subject to:

(7) $$S_{W}\left(g,\;g^{+}\right)-S_{W}\left(g,\;g^{-}\right)\geq 1-\xi ,\;\xi \geq 0,$$

where ||.||_Frob _denotes the Frobenius norm, and ξ is a slack variable. The intuition behind this update is to keep *W^i ^*close to *W^i-1 ^*from the previous iteration while minimizing the current loss. Here, "aggressiveness" parameter α controls the trade-off between the two objectives. This optimization problem can be solved by the Lagrange method, resulting in the following update:

(8) $$W^{i}=W^{i-1}+\tau_{i}V^{i},$$

where

(9) $$\tau_{i}=\text{min}\left\{\alpha ,\frac{l_{W^{i-1}}\left(g,g^{+},g^{-}\right)}{||V^{i}|{{|}^{2}}_{Frob}}\right\},$$

and

$$V^{i}={\left[\Phi {\left(g\right)}_{1}\left(\Phi \left(g^{+}\right)-\Phi \left(g^{-}\right)\right),\ldots ,\Phi {\left(g\right)}_{d}\left(\Phi \left(g^{+}\right)-\Phi \left(g^{-}\right)\right)\right]}^{T},$$

where Φ(*g*)*_i _*denotes the *i*-th element of Φ(*g*).

This learning procedure continues until a stopping condition is satisfied, and the corresponding *W *is returned. In practice, one can select the best *W *by using a heldout validation set: the accuracy is measured on the validation set and learning stops when the accuracy becomes saturated. As reported in [24], using this method to select *W *provides good generalization while reduces learning time.

### Estimating pairwise similarities between training genes

The algorithm described in the previous section requires training signals in forms of triplets (*g*, *g*^+^, *g*^-^). From the set of *d *genes with known annotations it is important to choose only right triplets so that genes *g *and *g*^+ ^are functionally similar while genes *g *and *g*^- ^are not. For cases in which each gene has a single function, selecting such a triplet is straightforward in that pairs of genes that share a function are more similar than pairs of genes with different functions. This leads to a simple strategy, in which one can select a gene *g*, find a gene with the same function as *g *to provide an instance of *g*^+^, then find a gene without that function to provide an instance of *g*^-^. In practice, however, a gene can have multiple functions or participate in multiple biological processes. Moreover, genes are often annotated with functions that form hierarchies, as in the case of GO or FunCat categories [25,26]. These properties make it more complex to quantify the functional similarity between gene pairs when choosing triplets for training.

A number of methods and metrics have been proposed to quantify the semantic similarity between GO terms (see [28] for a review). In this study, we use Resnik's measure [26] - one of the most stable and widely used similarity metrics for biomedical ontologies like GO [27] - to estimate functional similarities. Resnik defines the semantic similarity between a pair of GO terms *c*_1 _and *c*_2 _as the information content (IC) of their most informative common ancestor (MICA) according to the GO graph:

$$\text{Sim}\left(c_{\text{1}},c_{\text{2}}\right)\;=\text{ IC}\left(c_{\text{MICA}}\right)$$

The IC of a term is defined as the negative log of the probability that this term appears in a collection of gene annotations.

Since a gene can be annotated with multiple GO terms, we estimate the similarity between a pair of genes by combining the Resnik's measures of their annotations. There are several combination strategies including maximum, average, only exact matches, or sum of all pairs [28]. Here we adopt the best-match average combination method: we take only the best matched terms and estimate their average Resnik-based similarities. This combination method has been reported to give intuitive and stable results in several benchmarks [28]. In our experiments, Resnik's similarities between GO terms as well as similarities between gene pairs were computed by using the GoSemSim package [29].

### Selecting training triplets for learning function specific similarity measures

Using the learning procedure described in the previous section, for each functional category *C*, we estimate a parameter matrix *W_C_*. In the next step, *W_C _*will be used to construct a new association network that is specific for *C*. Note that, it is more computationally efficient to estimate and store a single functional network for all categories. However, because a gene can have multiple functions, such a single network may be insufficient to represent all the co-functionality relationships between genes. Maintaining one separate network for each function can provide more information to make accurate predictions.

#### Strategies for selecting training triplets

For a given functional category *C*, the following procedure is used to select a gene triplet for training. First, select at random a gene annotated with *C*, which will be *g*. Then, select at random another gene also annotated with *C*, which will be *g*^+^. Finally, select at random a negative gene *g*^-^that satisfies the following: (i) it is not annotated with *C *and any descendant term of *C *in the GO graph, and (ii) its similarity score with respect to *g *is lower than a threshold, where the similarity score *r *is estimated using the method described in the previous section. A threshold of 0.4 was used in our experiments, meaning that two genes were deemed to be not functionally related if their similarity score was lower than 0.4.

While *g *and *g*^+ ^are sampled uniformly, we consider three ways to sample *g*^-^ from those satisfying the two above conditions:

- Uniform sampling. This strategy considers all negative genes equally.

- Sampling a negative gene less relevant to *g *with a higher probability. The intuition behind this strategy is that we update the similarity function so that it returns true scores for the most dissimilar pairs of genes first. A negative gene is sampled with probability (1-*r*)/*z *where *r *is the Resnik-based similarity score between this gene and *g*, and *z *is the normalization factor.

- Sampling a negative gene more relevant to *g *with higher probability. This strategy attempts to first update the similarity function on borderline genes, that is, negative genes having similarity scores near the threshold. Specifically, a negative gene is sampled with probability *r*/*z*, where *r *is the similarity score between this gene and *g*, and *z *is the normalization factor.

We initialized *W^0^_C _*to an identity matrix, i.e. *W^0^_C _*= *I*, and used a validation set to select the best *W_C_*: the prediction accuracy was periodically measured on the validation set after a predefined number of iterations; learning stopped when accuracy became saturated and the corresponding *W_C _*was returned.

### Constructing new networks

Once similarity functions are learned, the next step is to construct new association networks, one per a GO term. For each gene *g_i_*(*i *= 1..*n*) from the annotated and unannotated gene sets, we create its feature vector using equation (1). Then, for each functional category *C*, we use similarity function $S_{W_{C}}$ with matrix *W_C _*to compute the *C *similarity score between genes *g_i _*and *g_j _*and use this score as the weight *a'_ij _*of the edge connecting the genes (note that we omit index C from *a'_ij _*for simplicity).

Because $S_{W_{C}}$ is not symmetric, i.e. $S_{W_{C}}\left(g_{i},g_{j}\right)$ and $S_{W_{C}}\left(g_{j},g_{i}\right)$ are not necessarily the same, we compute *a'_ij _*as follows:

(10) $$a_{i\;j}^{\prime }=\left(S_{W_{C}}\left(g_{i},g_{j}\right)+S_{W_{C}}\left(g_{j},g_{i}\right)\right)/2$$

To sparsify the newly constructed networks we keep only *k *connections with the largest weights for each node and remove the remaining connections.

### Inferring gene function

Given an association network $\text{A'}=\left\{a_{ij}^{\prime }\right\},i,j=1...n$, any of existing graph-based classification algorithms can be used to infer functions of unannotated genes. In this study, we use the semi-supervised learning algorithm by Zhou *et al. *[30] for this step.

Let ***y ***denote a label vector, each element *y_i _*of which represents the prior knowledge about gene *i *having (or not) the function of interest. We assign labels +1 to positive genes, that is, genes known to have the given function, and assign labels -1 to negative genes. Here, we consider a gene negative if it is not annotated with the given function and any of its descendants according to the GO graph. Following Mostafavi *et al. *[10], we assign a prior value $y_{i}=\frac{d^{+}-d^{-}}{d^{+}+d^{-}}$ for genes with unknown annotations, where *d*^- ^and *d*^+ ^are the numbers of negative and positive genes in the training set, respectively. This prior value is used to reflect the class imbalance nature of the gene function prediction problem, in which the number of negative genes is typically much larger than the number of positive genes.

The learning process consists of estimating a score *f_i _*∈[-1,1] for each gene g_*i*_. Once this score is estimated, the algorithm classifies the gene into having or not having the given function by thresholding the score. Score *f_i _*is obtained by minimizing the following objective function:

(11) $$\overset{n}{\underset{i=1}{\sum }}{\left(y_{i}-f_{i}\right)}^{2}+\sigma \overset{n}{\underset{i,j=1}{\sum }}a_{ij}^{\prime }{\left(f_{i}-f_{j}\right)}^{2}={\left(y-f\right)}^{T}\left(y-f\right)+\sigma f^{T}Lf,$$

where *D *is a diagonal matrix with and $d_{ii}=\sum_{j=1}^{n}a_{ij}^{\prime }$ and L=D-A' is the graph Laplacian matrix. This objective function has two terms: the first term constrains score *f_i _*not to change much from prior label *y_i_*, and the second term encourages adjacent nodes to have similar scores. Parameter σ trades off these two competing objectives. This optimization problem has the following solution:

(12) $$f={\left(I+\sigma L\right)}^{-1}y,$$

### Input networks

Up to this point, we assumed that the input combined network was given. In practice, one can obtain such a network by using any existing network integration method. In our experiments, we considered a very simple integration method in which the combined network is created by summing over individual networks, and all the networks have the same weight. We sparsified the networks by keeping only 50 edges with the largest weights for each node and removing the rest. The number of 50 edges for each node was chosen based on the results from [11].

### Datasets

We used two datasets in two species (yeast and human) to evaluate the effectiveness of the proposed method.

#### The yeast dataset

The yeast dataset is provided by Barutcuoglu *et al. *[21] and contains various genomic and proteomic data for 4524 yeast genes. There are four types of data: microarray data, transcriptions factor binding sites, protein-protein interactions, and co-localization of gene products in a cell. The interaction, co-localization, and binding site data are binary, and microarray data are real-valued. 105 GO terms selected from the *biological process *vocabulary of GO were used as labels to annotate the genes. To ensure consistency among the training labels, all annotations were up-propagated, that is if a gene is assigned to a term in the GO graph, it is also assigned to all ancestors of this term. This procedure was applied in all our experiments.

#### The human dataset

We used the human dataset provided by Mostafavi *et al. *[11]. This dataset contains various biological data collected from eight sources for 13281 human genes. The data include OMIM diseases associated with genes, domain compositions, protein interactions, transcriptional modification data, and gene expression data. Gene expression data are real numbers while the other data are binary. The genes in this dataset were annotated with terms from the *biological process *vocabulary of GO. The same procedure as used for the yeast dataset was applied to up-propagate the annotations. To guarantee the same experimental conditions, we followed the steps described in [11] to create individual networks for the eight data sources. For each dataset, we computed the association between a pair of genes as the Pearson correlation coefficient (PCC) of the two feature vectors representing these genes. We kept only positive PCC values and set negative ones to zeros. For protein interaction data, in addition to networks computed by using PCC, we also used the interaction networks directly.

## Results and discussion

We used 3-fold cross validation to evaluate the effectiveness of the proposed methodology (SLAN) in predicting GO functional classes for the two datasets and compared the results against those of two other methods (we could not use more than three folds because some GO terms had only three positive genes in the experimented datasets). The performance of each method under test was measured by computing the AUC score, which is the area under the receiver operating characteristic (ROC) curve. AUC is a measure of choice when assessing the performance of methods that returns continuous scores such as the method we use in the prediction step. An AUC score of 1 corresponds to perfect classification with negative examples successfully separated from positive ones, while random guessing results in an AUC score of 0.5. For each split of a dataset into training and test sets, we withheld 25% of the training set to use as a validation set for determining the stopping point of the learning algorithm. We computed AUC scores on the validation set every 2000 iterations, and stopped learning once the accuracy became saturated. In the following sections we report the AUC scores averaged over three folds.

All three sampling strategies, i.e. uniform sampling, sampling less relevant negative genes with higher probabilities, and sampling more relevant negative genes with higher probabilities yielded similar AUC scores but the third strategy was significantly faster than the first and second ones as it required fewer training iterations. In the following section we report results when the third sampling strategy was used.

### Results on the yeast dataset

#### Comparison with SW

In the first experiment, we compared our method (SLAN) with the SW method by Mostafavi and Morris [11], using the yeast dataset. SW is a fast network-based method that achieved leading prediction accuracy in a number of gene function prediction benchmarks [11,20]. The SW algorithm integrates multiple networks, each of which is computed from a dataset, into a single combined network that it then uses to infer gene function. In SW, a combined network is a weighted linear combination of individual networks. SW formulates the network integration problem as a linear regression problem and simultaneously optimizes the weights over a group of related functional categories. Because our method and SW use the same algorithm to predict gene function from an association network, the difference in network quality is the only factor that makes the accuracy of the two methods different. Thus, the superiority in prediction accuracy of either method would mean that this method produces networks of better quality. We used the Matlab implementation of SW provided by its authors with all parameters set to default values.

**Figure 1:**
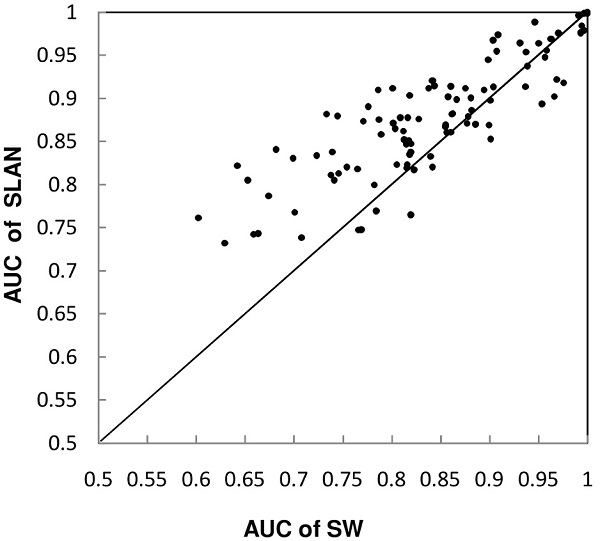
**AUC score comparison between SW and SLAN on the yeast dataset**. Each point represents a GO term, showing AUC scores for SW and SLAN on the x-and y-axes respectively. Points above the diagonal correspond to accuracy improvement by SLAN.

The AUC scores of SW and SLAN for 105 GO terms are shown and compared in Figure 1. Out of 105 GO terms, SLAN achieved higher AUC scores than SW for 77 GO terms, and SW scored higher or equally in the remaining 28 cases. Over all 105 GO terms, SLAN achieved an average AUC value of 0.882, and SW achieved an average AUC value of 0.847. The result of a Wilcoxon signed rank test showed that the difference in AUC scores between SLAN and SW was significant, with *p-value *= 1.36 × 10^-8^.

To understand the behavior of the algorithm, we inspected intermediate results of the learning step. We found that most GO terms, for which SLAN achieved lower accuracy than SW, were associated with a similarity learning step that stopped immediately because it decreased the prediction accuracy. An explanation for this result is that the similarity functions cannot capture all the functional associations between genes, especially when these associations are complex. A typical situation, in which such complexity arises, is when each gene has multiple functions which themselves are related. It is also possible that the use of the graph-based algorithm that predicts gene labels from a network may inherently cause early cessation of the similarity learning step. This semi-supervised algorithm relies on the global structure of the network, which means that the solution depends on every association, including associations between negative genes. Since the similarity learning procedure ignores such associations, it can decrease accuracy in some functional classes. In such cases, further learning would lead to undesired effects, which can be prevented by early stopping with the help of a held-out set.

#### Comparison with hierarchical decision tree ensembles

In the second experiment, we compared our method with CLUS-HMC-ENS [31] - a recently proposed method that does not rely on association networks, thus represents another class of gene function prediction methods. CLUS-HMC-ENS takes as input vector representations of genes and classifies genes into functional groups by learning an ensemble of decision trees. The trees are "hierarchical" in the sense that they exploit the hierarchy nature of GO and each tree can make predictions for all classes at once. We used the implementation of this ensemble method provided by its authors. CLUS-HMC-ENS was run with all default settings - the settings that provided the best performance in previous experiments.

**Figure 2:**
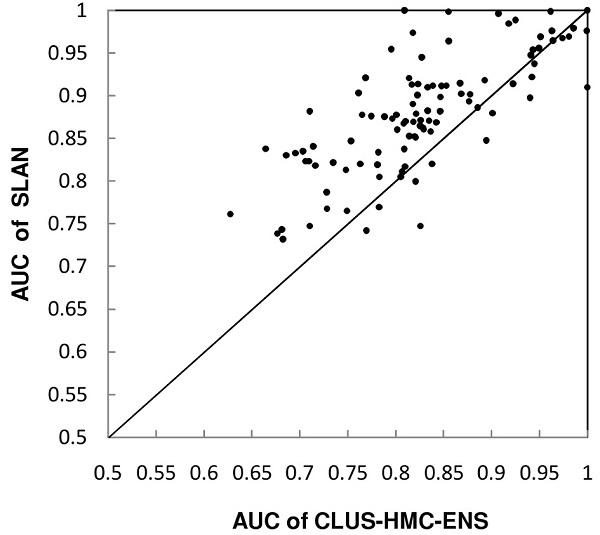
**AUC score comparison between CLUS-HMC-ENS and SLAN on the yeast dataset**. Each point represents a GO term, showing AUC scores for CLUS-HMC-ENS and SLAN on the x- and y-axes. Points above the diagonal correspond to accuracy improvement by SLAN.

Figure 2 plots the AUC scores of CLUS-HMC-ENS against those of SLAN for the yeast dataset. Over all 105 GO terms, the average AUC score of CLUS-HMC-ENS was 0.831, which was lower than those of both SLAN and SW methods. SLAN achieved higher AUC scores than the tree-based ensemble method for 85 out of 105 GO terms. Overall, SLAN performed significantly better than CLUS-HMC-ENS in terms of AUC, according to a Wilcoxon signed rank test (*p-value *= 2.08 × 10^-10^).

### Results on the human dataset

**Figure 3:**
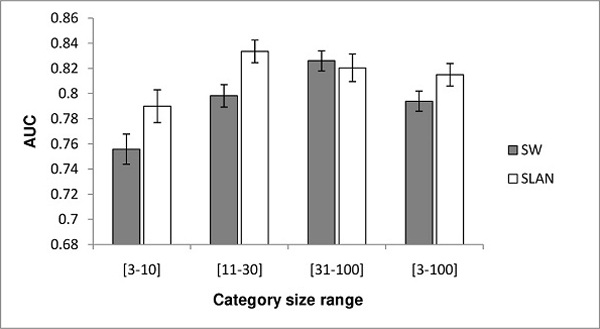
**AUC score comparison between SW and SLAN on the human dataset**. AUC scores are grouped into four evaluation categories corresponding to four groups of GO terms with [3-10], [11-30], [31-100], and [3-100] (overall) positive genes. For each category, grey bars and white bars show average AUC scores of SW and SLAN respectively. Error bars show the standard errors.

In the next experiment, we evaluated and compared SLAN and SW on the human dataset. This dataset contains more GO terms than the yeast dataset, and the number of positive genes annotated to a term ranges from three to 100. Because the prediction accuracy of a classification algorithm depends on the size of training data, we grouped the results into four categories corresponding to four groups of GO terms with [3-10], [11-30], [31-100], and [3-100] (overall category) positive genes, as done in [20]. In Figure 3, we summarize the average AUC scores of each method for each of the four evaluation categories. As shown, SLAN scored lower than SW for GO terms with [31-100] positive annotations but achieved higher average AUC scores than SW in [3-10] and [11-30] categories. The results also show that SLAN produced more accurate predictions than SW for the overall category [3-100], which included all the GO terms used in the dataset.

The fact that different methods achieve the best performance in different evaluation categories, as observed in this experiment, was also reported in [12], suggesting that there is rarely a single method that delivers the best result in all situations. A possible way to achieve superior performance in all prediction scenarios is to use a combination of different methods. The superiority of SLAN over SW in categories with small number of positive annotations ([3-10] and [11-30] categories) shows its appropriateness for scenarios when few positive training examples are available. Despite the fact that SLAN achieved lower AUC value than SW in one of three individual categories, the superior accuracy of SLAN over all the GO terms used (shown in [3-100] overall category) demonstrates its ability to improve networks produced by a simple network integration method with fixed and equal network weights.

### Prediction accuracy bias over the GO functional groups

**Figure 4:**
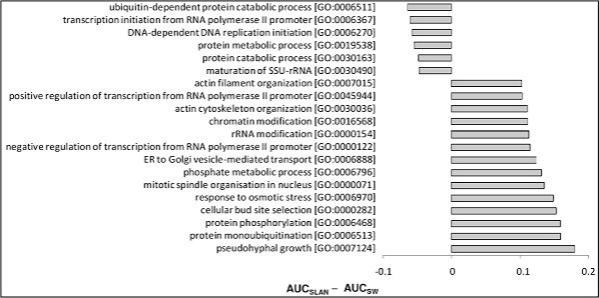
**List of GO terms with the largest differences in predictability, as determined by SW and SLAN on the yeast dataset**. GO terms corresponding to the largest absolute values of ΔAUC = max(AUC_SLAN_, 0.5) - max(AUC_SW_, 0.5) are shown on the left, and the bars show the values of ΔAUC on the right. For brevity, the maximum number of terms with negative and positive ΔAUC was set to 6 and 14, respectively.

**Figure 5:**
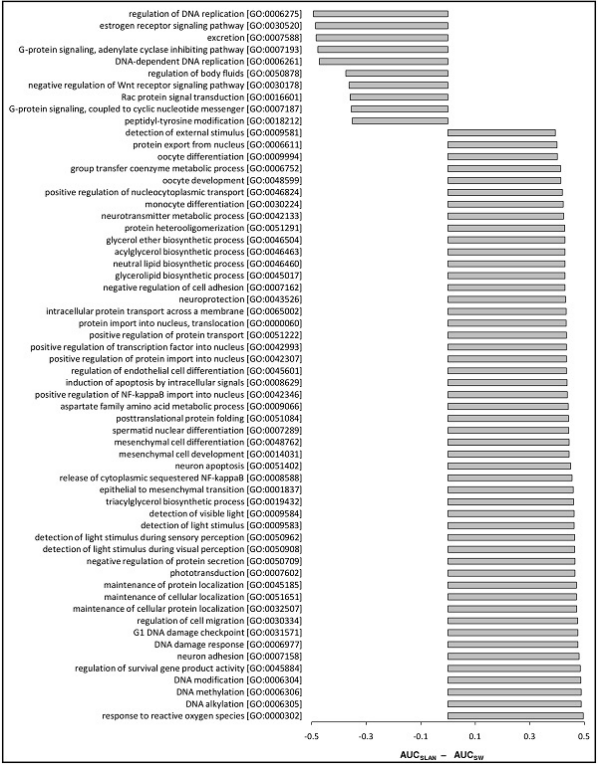
**List of GO terms with the largest differences in predictability, as determined by SW and SLAN on the human dataset**. GO terms corresponding to the largest absolute values of ΔAUC = max(AUC_SLAN_, 0.5) - max(AUC_SW_, 0.5) are shown on the left, and the bars show the values of ΔAUC on the right. For brevity, the maximum number of terms with negative and positive ΔAUC was set to 10 and 50, respectively.

The results above revealed differences in the performances of the methods tested across GO terms. For some GO terms, the proposed method showed better prediction accuracy, whereas for other GO terms, it gave less accurate predictions than other methods. Given this observation, we asked on which GO terms there are the largest variations in prediction accuracy between our and other methods. In our investigation, AUC was used as the relative measure of performance for comparing SLAN and SW. We calculated the difference in the AUC scores between SLAN and SW for each GO term, sorted GO terms in order of increasing difference, and examined those GO terms with the largest AUC differences. Because AUC = 0.5 corresponds to a random guess, we set the minimum AUC score for each method to 0.5 by using max(AUC, 0.5) in the comparison, that is, ΔAUC = max(AUC_SLAN _, 0.5) - max(AUC_SW_, 0.5). The lists of the GO terms with the largest |ΔAUC| for the yeast and human datasets are given in Figures 4 and Figure 5, respectively. The figures show large differences in prediction accuracy for some GO terms, suggesting that each prediction method is more appropriate for certain functional groups. For example, on the human dataset, the term "response to active oxygen species [GO:0000302]" was accurately predicted by SLAN, and poorly predicted by SW. The difference in the AUC corresponding to this term was nearly 0.5. In contrast, the term "regulation of DNA replication [GO:0006275]" was accurately predicted by SW, but poorly predicted by SLAN. We also observed that the largest |ΔAUC| from the yeast dataset were smaller than those from the human dataset, mainly because the subset of GO terms used in the first dataset was smaller and less diverse [21].

### Computational time

As mentioned above, the third sampling strategy required fewer training iterations than the other two in all experiments, suggesting that one should optimize the similarity function on more difficult training triplets first. In this section, we report the computational time when the third sampling method was used. On average, training of the similarity functions over all 105 functional classes on the yeast dataset using a uniformly weighted network input saturated after 0.9 million iterations (triplets) and took 15 minutes on a single CPU of a modern PC running Linux. In contrast, CLUS-HCM-ENS took more than 3 hours to learn an ensemble of 50 decision trees on the same data set. Since CLUS-HCM-ENS is much faster than other classifier-based methods that create one binary classifier for each functional class [29], these results suggest that our method compares favorably with classifier-based gene prediction algorithms in terms of speed. On the human dataset, similarity training stopped after 9.5 million iterations on average and took less than two and a half hours. The running time on the human dataset indicated that although SLAN was slower than some network learning approaches, such as the ones proposed in [9,11], its computational complexity is acceptable, even for gene function prediction in large mammalian genomes.

**Figure 6:**
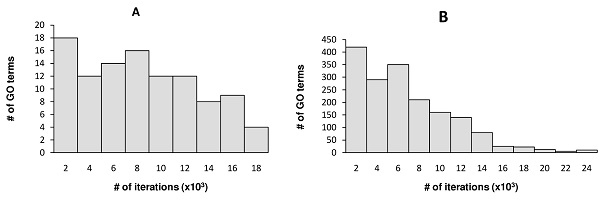
**Distribution of training iterations across groups of GO terms**. GO terms with the same number of iterations were grouped together. The x-axis shows the number of iterations before the similarity learning algorithm stopped. The y-axis shows the number of GO terms in each group. The left panel (A) includes results from the yeast benchmark. The right panel (B) includes results from the human benchmark.

Figure 6 shows the numbers of learning iterations for different GO term groups (each group consisted of GO terms with the same number of training iterations). As shown, less than 16x10^3 ^iterations were required for most GO terms before learning stopped. Despite the larger number of genes in the human dataset, the average training time for a GO term on this dataset was comparable to that of the yeast dataset.

## Conclusions

In this study, we propose a new method for optimizing functional association networks that are used in predicting gene function. While existing approaches focus on constructing combined networks from individual ones, the proposed method focuses on improving combined networks already constructed. By using similarity learning algorithms originally developed for multimedia search applications, our method can produce new association networks with improved prediction accuracy. In experiments with yeast and human, the networks optimized by our method yielded significant improvements in terms of AUC scores, and the learning time was acceptable even for the large human genome. The results show that it is possible and useful to optimize combined networks before using these networks for prediction, and this optimization step can be performed by learning appropriate similarity measures from data. The proposed method can be applied to networks produced by any integration algorithm, thus provides a good complement for existing approaches. Other applications of similarity learning, for example in computing network weights during the integration phase, will be investigated in future work.

## Competing interests

The authors declare that they have no competing interests.

## Authors' contributions

TMP conceived of the study, participated in its design, and drafted the manuscript. NPN participated in study design, implemented the experiments, analyzed the data, and helped to draft the manuscript. All authors read and approved the final manuscript.

## Declarations

The publication costs for this article were funded by the National Foundation for Science and Technology Development of Vietnam.

This article has been published as part of *BMC Genomics *Volume 14 Supplement S4, 2013: Selected articles from the IEEE International Conference on Bioinformatics and Biomedicine 2012: Genomics. The full contents of the supplement are available online at http://www.biomedcentral.com/bmcgenomics/supplements/14/S4.
